# Case Report: A New Entity: Multiple Differentiated Variant of Papillary Thyroid Carcinoma With Advanced Clinical Behavior

**DOI:** 10.3389/fendo.2021.654638

**Published:** 2021-04-07

**Authors:** Jing Yang, Rixiang Gong, Yu Ma, Jun Gao, Zhihui Li, Jingqiang Zhu, Yanping Gong

**Affiliations:** ^1^Thyroid and Parathyroid Surgery Center, West China Hospital, Sichuan University, Chengdu, China; ^2^Department of Toxicological Inspection, Sichuan Center for Disease Control and Prevention, Chengdu, China

**Keywords:** PTC, single tumor, morphological patterns, multiple differentiated variant, entity

## Abstract

There are many histological morphological types of papillary thyroid carcinoma (PTC), but the most frequently seen types are conventional. A single PTC commonly has a conventional and/or a variant morphological pattern. PTC with multiple (more than two) well-differentiated morphological patterns are extremely rare. We herein report the rare case of a 48-year-old male with initial diaphragmatic, pancreatic, and liver tumors from PTC. Then, the PTC was discovered following resection of these tumors, an ultrasound-guided fine-needle aspiration (US-FNA) cytology of a huge mass in the thyroid’s left lobe revealed a PTC. After postoperative recovery, physical and ultrasound examinations identified an irregular large nodule in the thyroid’s isthmus and left lobe, several swollen lymph nodes in the left neck, a mass in the left gluteus maximus, and several masses in both the bilateral parotid and salivary regions. The US-FNA’s pathological examination confirmed metastatic PTCs in the left gluteus maximus and bilaterally in the parotid and salivary glands. An 18-fluorodeoxyglucose positron-emission tomography and computed tomography scan revealed abnormal uptakes in numerous locations (e.g., thyroid’s isthmus and left lobe, bilateral parotid gland, and subcutaneous tissues). The patient underwent palliative therapy—including total thyroidectomy, bilateral central neck dissection, left lateral neck dissection, and excision of the bilateral parotid and salivary glands. A whole-body scan post-therapeutic radioactive iodine ablation revealed exclusive thyroid bed uptake. The patient subsequently underwent thyroid stimulating hormone (TSH) repression therapy and chemotherapy with lenvatinib, and thereafter achieved stable clinical conditions. Further histopathological analysis of the PTC revealed multiple differentiated morphological patterns in the single tumor located in the isthmus and left lobe of the thyroid, and in some metastatic lesions. Different metastatic lesions also presented different morphological patterns of PTC. In conclusions, we identified a new entity of PTC as a multiple differentiated variant of PTC (MDV-PTC) with an aggressive clinical nature.

## Background

Papillary thyroid carcinoma (PTC) is the most common thyroid carcinoma and has many histological morphological variants, but the conventional type is commonly observed. The variants of PTC as classified by the World Health Organization (WHO) 2017 guidelines are: papillary microcarcinoma, encapsulated, follicular, diffuse sclerosing, tall cell, columnar cell, cribriform-morular, hobnail, fibromatosis/fasciitis-like stroma, solid/trabecular, oncocytic, spindle cell, clear cell, and Warthin-like (1). The follicular variant of PTC is the most common after conventional PTC (2), and other variants, such as diffuse sclerosing, tall cell, columnar cell, and hobnail variant are less often observed. In addition, conventional PTC usually has favorable clinicopathologic features and an excellent prognosis. However, some variants of PTC—such as diffuse sclerosing, tall cell, columnar cell, hobnail variant, and solid variants—behave more aggressively than conventional PTC (3, 4). In a single tumor, PTC commonly presents as conventional and/or a variant morphological pattern. PTC with multiple (more than two) well-differentiated morphological patterns is extremely rare. We herein report a 48-year-old male who presented with a single thyroid tumor composed of conventional PTC morphology with co-existent multiple variant patterns of PTC, as well as cervical lymph node metastases and multiple distant metastases of multiple morphological patterns of PTC.

## Case Presentation

A 48-year-old male was referred to the Pancreas Surgery Department of West China Hospital, Sichuan University for a mass of the pancreatic body and a nodule of the right liver as identified on abdominal ultrasound examination. The patient had not any significant findings in his past medical history except excision of superficial lipomyoma and pancreatitis. He had a history of smoking, but not drinking or drug abusing. He neither had exposure to radiation. Family history revealed the presence of lung cancer in his father. Subsequently, the patient underwent resection of a firm irregular masse (1.5 × 1.0 × 0.7 cm) located in the diaphragm, a firm irregular masse (measuring 2.5 × 2.0 × 1.5 cm) in pancreatic body, and three firm irregular masses (diameter: 0.5–0.8cm) on the surface of the liver on January 31, 2018. Pathological examination and immunohistochemical analyses of these excised tissues revealed metastatic PTCs. A subsequent cervical ultrasound examination revealed a huge mass (8.1 × 5.1 × 4.1 cm) in the left lobe of the thyroid, and an ultrasound-guided fine-needle aspiration (US-FNA) cytology revealed PTC.

**Figure 1:**
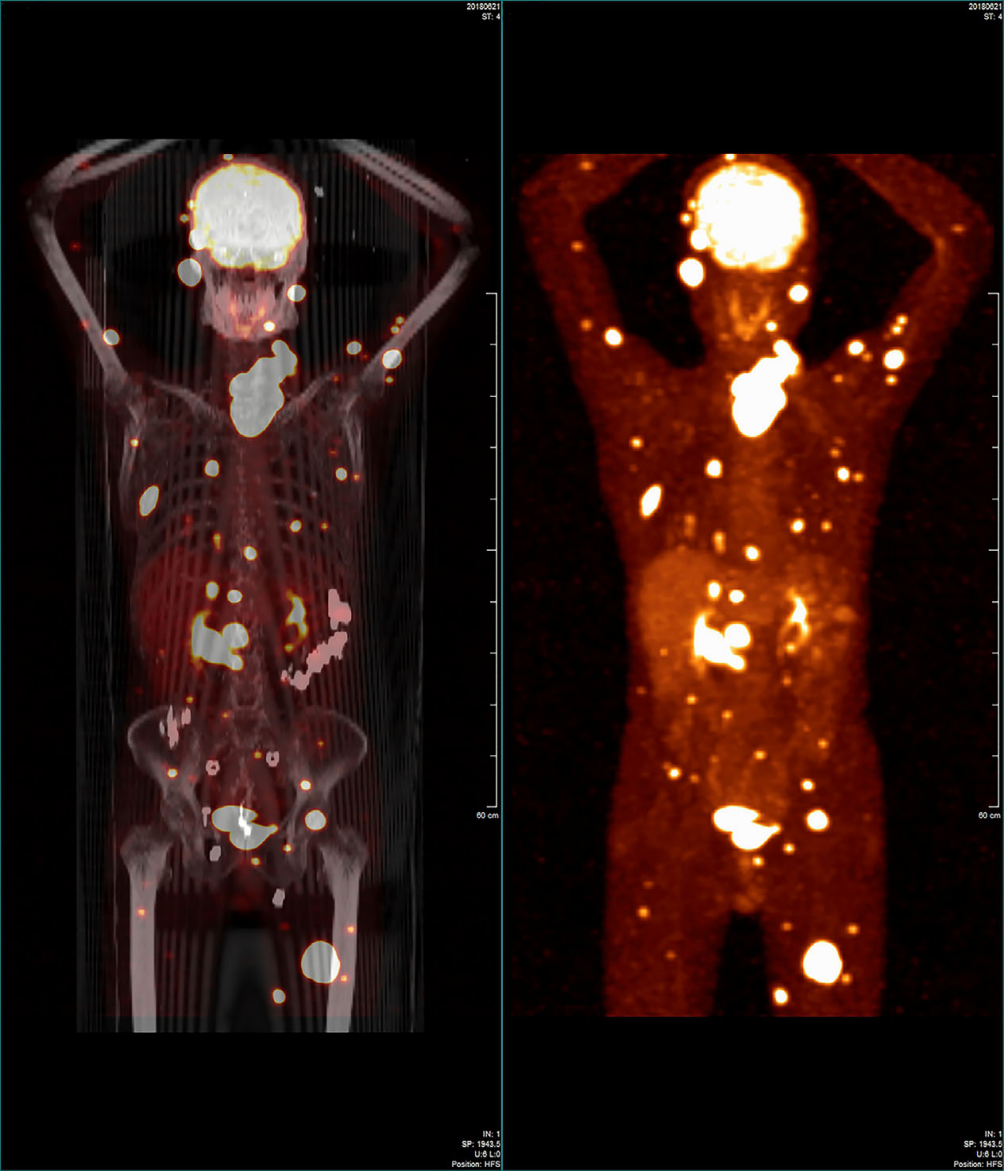
Whole-body 18-fluorodeoxyglucose positron-emission tomography/computed tomography image showing that many regions in the patient’s body had widespread abnormal uptake.

The patient was referred to the Thyroid and Parathyroid Surgery Center of our institution for evaluation for thyroid surgery in June 2018 following a postoperative recovery period of more than four months. A physical examination showed an irregular large nodule in the isthmus and left lobe of the thyroid, a swollen lymph node in the left cervical lateral compartment, and a mass in the right parotid gland. An ultrasound reexamination revealed the following findings: a very large hypoechoic mass (7.3 × 5.9 × 5.1 cm) in the isthmus and entire left lobe of the thyroid, multiple hypoechoic nodules (about 0.2–0.3 cm) in the right lobe of the thyroid, several swollen lymph nodes in the left cervical lateral compartment, several masses in the bilateral parotid region, several nodules in the bilateral salivary region, and a mass in the left gluteus maximus. US-FNA’s histological results and immunohistochemical analyses confirmed the presence of metastatic PTCs bilaterally in the parotid gland, and bilaterally in the salivary gland. Both an 18-fluorodeoxyglucose positron-emission tomography and computed tomography scan were ordered for further evaluation and identification of possible occult metastases. The first revealed abnormal uptakes in the following locations: isthmus and left lobe of the thyroid (maximum standardized uptake value [max SUV]:25.22), bilaterally in the parotid gland (left max SUV:16.02, right max SUV:22.51), left salivary gland (max SUV:8.7), left cervical region (max SUV:15.6), bilaterally in the lungs (max SUV:13.41), pancreatic head (max SUV:17.12), right kidney (max SUV:8.71), multiple cones and ribs (max SUV:17.50), and muscles and subcutaneous tissues (max SUV:26.96) (**Figure 1**).

The patient underwent palliative surgical therapy. Specifically, on July 2, 2018, the patient received a total thyroidectomy, bilateral central neck dissection, left lateral neck dissection, and bilateral excision of the parotid and salivary glands. A firm irregular tumor measuring 10.0 × 7.0 × 5.0 cm was observed intraoperatively. The tumor mass replaced the isthmus and left lobe of the thyroid. Additionally, it extended into the tracheoesophageal region and deepened into the superior mediastinum, invading the anterior cervical skeletal muscle. Furthermore, the tumor encapsulated and infiltrated the left recurrent laryngeal nerve and slightly infiltrated the left tracheal surface. Several swollen cervical lymph nodes were identified. The largest one measured 4.0 cm. Bilaterally in the parotid and salivary regions, there were many masses ranging from 0.5–3.5 cm. Of note, some of the masses invaded the facial nerve. Histological examination was performed postoperatively on the paraffin-embedded specimen. The results revealed PTC with multiple regional lymph node metastases, and distant parotid and salivary gland metastases. In addition, *BARF^V600E^* and *TERT* promoter mutation (C288T) were identified by next-generation sequencing in the primary thyroid tumor, the pancreatic and cervical lymph node metastases.

Postoperatively, the patient underwent TSH repression therapy (TSH < 0.10 mU/L) with oral administration of sodium levothyroxine (Euthyrox). Oral sodium levothyroxine treatment was suspended in August 2018 in preparation for radioactive iodine ablation therapy. RAI ablation therapy with 200 millicurie of I-131 was administered after two weeks. A whole-body scan following therapeutic RAI ablation showed uptake only in the thyroid bed and no uptake in any of the metastasizing lesions. Subsequently, the patient underwent continuous TSH repression (TSH < 0.10 mU/L) therapy with oral sodium levothyroxine. Additionally, he was treated with lenvatinib chemotherapy from December 2018. Computed tomography revealed that after more than 2 years of treatment, the number of metastatic lesions decreased. Additionally, serum thyroglobulin decreased to 15.84~50.60 ug/L (from 313.60 ug/L) as of December 31, 2020. The patient is currently alive with no apparent symptoms. Most of the above medical details have been presented in an other publication regarding the same case by Jing Y et al. (5), which can provide other more detailed medical information.

**Figure 2:**
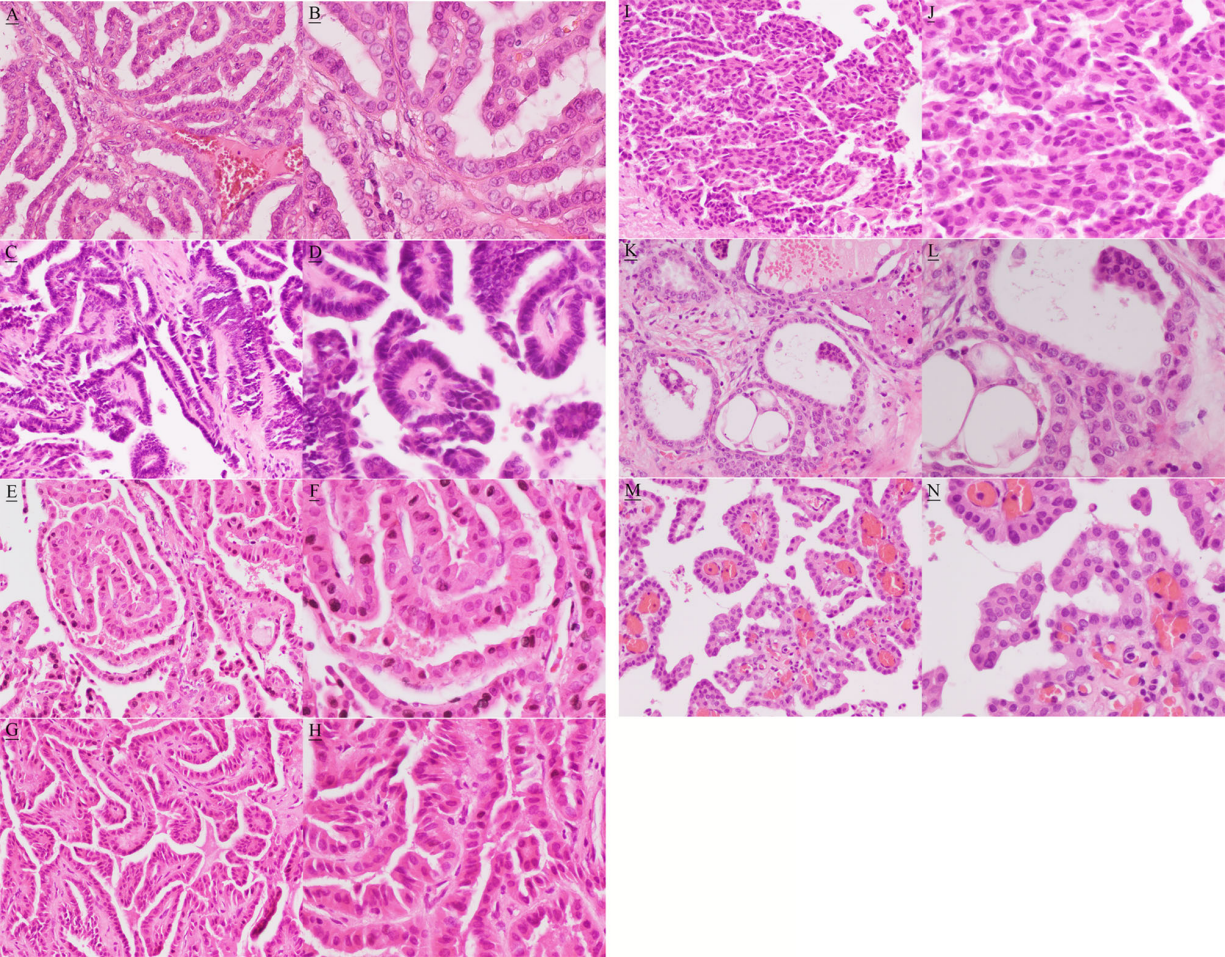
Hematoxylin and eosin image showing conventional (**A**, magnification, x200; **B**, magnification, x400 ), columnar (**C**, magnification, x200; **D**, magnification, x400 ), tall cell (**E**, magnification, x200; **F**, magnification, x400 ), cribriform-morular (**G**, magnification, x200; **H**, magnification, x400 ), solid/trabecular (**I**, magnification, x200; **J**, magnification, x400 ), follicular (**K**, magnification, x200; **L**, magnification, x400 ), and hobnail (**M**, magnification, x200; **N**, magnification, x400 ) morphological patterns.

The postoperative formalin-fixed, paraffin-embedded tissue blocks were selected and hematoxylin–eosin (HE) stained slides were cut for further identification of histopathological subtypes of PTC. The paraffin sections were read after HE staining by Jun Gao’s pathology team. These histopathological results showed that there were multiple differentiated morphological patterns in different sections and also in some of the same sections (**Figure 2**). Conventional morphological patterns of PTC were found in all sections. In addition, there was co-existence of columnar cell, tall cell, cribriform-morular, and solid/trabecular morphological patterns of PTC in the single tumor located in the isthmus and left lobe of the thyroid. There was co-existence of columnar, tall cell, and hobnail morphological patterns of PTC in cervical lymph nodes, and co-existence of columnar and tall cell morphological patterns of PTC in masses of the parotid and salivary glands. There was also co-existence of follicular and columnar morphological patterns of PTC in the single pancreatic mass, as well as co-existence of follicular, tall cell, and hobnail morphological patterns of PTC in masses of liver. Additionally, follicular and tall cell morphological patterns of PTC coexisted in a single mass of liver. There were columnar morphological patterns of PTC in a diaphragmatic mass. **Table 1** summarizes these results.

**Table 1:** Variant pathological patterns of PTC in different lesions.

Location of masses	Variant pathological patterns of PTC
Primary tumor	Columnar, tall cell, cribriform-morular, and solid/trabecular patterns
Cervical lymph nodes	Columnar, tall cell, and hobnail patterns
Parotid	Columnar and tall cell patterns
Salivary	Columnar and tall cell patterns
Pancreas	Follicular and columnar patterns
Liver	Follicular, tall cell, and hobnail patterns
Diaphragm	Columnar pattern

This case report was approved by the Institutional Review Board of West China Hospital of Sichuan University, and the patient has provided written informed consent for publicly publishing the case details and accompanying pictures.

## Discussion

With the exception of a conventional morphological pattern, only a variant morphological pattern of PTC is generally present in a single tumor. A single tumor with co-existing multiple variant morphological patterns of PTC is rare. Schopper HK et al. reported that a single thyroid tumor showed a combination of conventional PTC, follicular variant of PTC, clear cell variant of PTC, columnar cell variant of PTC, and poorly-differentiated thyroid carcinoma (PDTC) (6). In our present case, the single tumor showed multiple well-differentiated morphological patterns, including conventional, tall cell, columnar cell, cribriform-morular, and solid/trabecular morphological patterns. According to the present case and that reported by Schopper HK et al. (6), the entity may have multiple differentiating potential. Of course, it is possible that the multiple morphological patterns of PTC coexisted in initiation as a possibility of a supposed collision tumor. However, we think that the possibility of a collision tumor is less likely for five morphological components in a single primary tumor. In the present case, PDTC was not observed in the primary tumor. Most PDTCs arise from a follicular or PTC (7–10), and some PDTCs are *de novo* (11). Thus, our case may stand a good chance of dedifferentiation into PDTC.

In our present case, besides a single primary tumor presenting multiple well-differentiated variant carcinomas, some single metastatic foci also showed multiple variant carcinomas. The metastatic focus of the pancreas showed co-existence with columnar and follicular variant patterns of PTC, and one metastatic focus of the liver showed co-existence with tall cell and follicular variant patterns of PTC. The case reported by Schopper HK et al. showed that metastatic papillary clear cell carcinoma and columnar cell carcinoma were in juxtaposition in a lymph node deposit (6). However, the follicular variant pattern of PTC was not observed in the primary tumor. Furthermore, the hobnail variant pattern of PTC, which presented in a metastatic lymph node and a liver mass, was also not observed in the primary tumor. This indicates that metastatic lesions may also have multiple differentiating potential by imitating primary tumor behavior, thereby making the possibility of collision tumor less likely. Of course, it is possible that the follicular and hobnail variant patterns of PTC, which may be left out in examining the primary thyroid tumor, directly metastasize to lymph nodes and/or distant locations.

The case by Schopper HK et al. showed vascular invasion and extensive lymph node involvement of the primary tumor (6). Our case presented a more aggressive clinical behavior. The primary tumor has obvious extrathyroidal extension, invading the anterior cervical skeletal muscle, the left recurrent laryngeal nerve, and the left tracheal surface. Additionally, the patient had extensive cervical lymph node metastases and multiple distant metastases (including lung, bone, and other rare distant metastases). The aggressive clinical behavior may be relative to aggressive variants of PTC. Because the primary tumor contains multiple aggressive variant patterns of PTC, such as the columnar variant, tall cell variant and solid/trabecular variant (3, 4), and the histological morphological results of lymph node metastases and distant metastases were mainly columnar and tall cell variant patterns of PTC, co-existing multiple aggressive variant patterns of PTC may contribute to more aggressive behavior, which results in extrathyroidal extension, lymph node metastases, and early multiple distant metastases. The patient may have a poor prognosis based on these aggressive clinicopathological features. Published meta-analyses performed on PTC have shown that co-existent *BRAF^V600E^* and *TERT* promoter mutations have a synergistic effect on poor clinical outcomes (12, 13). In this case, *TERT* promoter and *BRAF^V600E^* mutations were simultaneously detected in the primary tumor, which also may contribute to a poor prognosis.

From our point of view, this present case is a distinct new entity and extremely rare. We defined the new rare entity as multiple differentiated variant of PTC (MDV-PTC). To the best of our knowledge, we are the first to define MDV-PTC. The diagnosis of MDV-PTC should meet the following criteria: 1) Diagnosis of PTC by conventional criteria, 2) two or more than two different morphological growth patterns existing in a single tumor, except for conventional PTC pattern. Some other variants of PTC, which were defined according to percentage of growth pattern, may present two growth patterns. For instance, the hobnail variant of PTC is defined by > 30% of cell with hobnail features, and 3) absence of poorly-differentiated carcinoma patterns. The fourth edition (2017) of the WHO Classification of Tumors of Endocrine Organs reclassified PDTC as an independent class (1). Also, PDTC is the absence of the conventional nuclear features of PTC according to the histopathological diagnostic criteria for PDTC listed in the Turin consensus proposal (14). Of note, the MDV-PTC should be deemed as an aggressive variant for having aggressive clinicopathological features, including large tumor size, wide extrathyroidal extension, and the presence of lymph node metastases and multiple distant metastases.

## Concluding Remarks

We report an extremely rare new entity of PTC, and we firstly define the new entity as MDV-PTC. The MDV-PTC has an aggressive clinical behavior. However, additional future studies are needed to help us recognize the new entity.

## Data Availability Statement

The raw data supporting the conclusions of this article will be made available by the authors, without undue reservation.

## Ethics Statement

The studies involving human participants were reviewed and approved by The Institutional Review Board of West China Hospital of Sichuan University. The patients/participants provided their written informed consent to participate in this study. Written informed consent was obtained from the individual(s) for the publication of any potentially identifiable images or data included in this article.

## Author Contributions

JY, RG and YG managed the case. JY and YM collected patient data and image. JG designed method and did histopathological analysis. JY and YG wrote the manuscript. RG, ZL, and JZ conducted the study and revised the manuscript. All authors contributed to the article and approved the submitted version.

## Funding

This study was supported by grants from the Department of Sichuan Province, Science and Technology Support Program (grant no. 2018SZ0215).

## Conflict of Interest

The authors declare that the research was conducted in the absence of any commercial or financial relationships that could be construed as a potential conflict of interest.

## Abbreviations

PTC, Papillary thyroid carcinoma; WHO, Word Health Organization; US-FNA, Ultrasound-guided fine-needle aspiration; TSH, Thyroid stimulating hormone; HE, Hematoxylin-eosin; PDTC, Poorly-differentiated thyroid cancer; MDV-PTC, Multiple differentiated variant of PTC.
